# Spatial tracking of individual fluid dispersed particles via Raman spectroscopy

**DOI:** 10.1038/s41598-020-71253-x

**Published:** 2020-09-01

**Authors:** Benjamin Thomas Hogan, Jennifer O’Dowd, Joaquin Faneca Ruedas, Alexander Baranov, Anna Baldycheva

**Affiliations:** 1 Department of Engineering, University of Exeter, Exeter, UK; 2 EPSRC Centre for Doctoral Training in Metamaterials, University of Exeter, Exeter, UK; 3 Department of Information Technology and Electrical Engineering, University of Oulu, Oulu, Finland; 4 Department of Engineering, University of Cambridge, Cambridge, UK; 5 Faculty of Photonics and Optoinformatics, ITMO University, Saint Petersburg, 197101 Russia

**Keywords:** Nanoscale materials, Two-dimensional materials, Optical spectroscopy, Raman spectroscopy, Characterization and analytical techniques, Raman spectroscopy

## Abstract

We demonstrate a method for the spatial tracking of individual particles, dispersed in a fluid host, via Raman spectroscopy. The effect of moving a particle upon the intensity of different bands within its Raman spectrum is first established computationally through a scattering matrix method. By comparing an experimental spectrum to the computational analysis, we show that the position of the particle can be obtained. We apply this method to the specific cases of molybdenum disulfide and graphene oxide particles, dispersed in a nematic liquid crystal, and contained within a microfluidic channel. By considering the ratio and difference between the intensities of the two Raman bands of molybdenum disulfide and graphene oxide, we demonstrate that an accurate position can be obtained in two dimensions.

## Introduction

Dispersions of nanoparticles are becoming a ubiquitous element of everyday life, and are the subject of intensive scientific investigation. Applications have been developed spanning the breadth of: pharmaceuticals such as cancer treatment^1^ and drug delivery systems^2^; antibacterial and anti-fouling coating materials^3^; improved cosmetics formulations^3^; stronger construction materials^4^; and numerous other technologies^5–10^, representing a huge expenditure of human resources and effort. Furthermore, nanoparticles of scientific interest are not uniquely synthetic. Biological systems-including the human body-rely heavily on a variety of nanoparticles to function healthily and efficiently^1^. The ever-expanding pool of nanoparticle applications holds great potential for researchers to further exploit. As applications advance, there is concurrently an increase in the demand for methods by which the properties of nanoparticles can be both rapidly and accurately obtained^1,10^. One method that is widely applied to particle analysis is Raman spectroscopy^11^, which can give information on the chemical make-up of an analyte particle^11–14^. Additionally, the use of microfluidic systems to either enable rapid throughput characterisation, or to enable particle synthesis, is commonplace^15–18^. Such microfluidic systems essentially consist of a channel, which in two dimensions reduces to a cavity. By containing particles in a microfluidic cavity during spectroscopic analysis, we introduce additional contributions to the spectral signature owing to the properties of the cavity itself^19–21^.

It is well-known that cavities have associated modes, relating to the resonant wavelengths of the cavity^22,23^. This resonance gives an intrinsic background to any spectra obtained within the cavity. Cavity resonances can also couple to the vibrational resonances of the analyte particle, manifesting as shifts in the peak positions in the spectra obtained^24^. The electromagnetic field intensity varies across the cavity due to the superposition of different resonances^25^, and hence spectral intensity is strongly dependent on position^21^. Without accounting for these effects, there is a distinct possibility of incorrectly analysing a spectrum^20^. For example, at a large scale, concentrations could be under- or over-estimated as intensities of peaks are affected depending on position. Properties such as the number of layers of a 2D material, which can be established from the precise Raman shift and intensity ratios, are also prone to incorrect proscription^24^. However, the inconvenience of the additional analysis requirements is potentially offset by some benefits. Chief among these, the fact that the position of a particle within the cavity strongly affects the intensity of the Raman spectrum suggests the possibility that the position can in turn be established via the spectrum. In this work, we aim to demonstrate that the variation in the Raman signal can be used to accurately predict particle positions in two dimensions.

## Results and discussion

**Figure 1:**
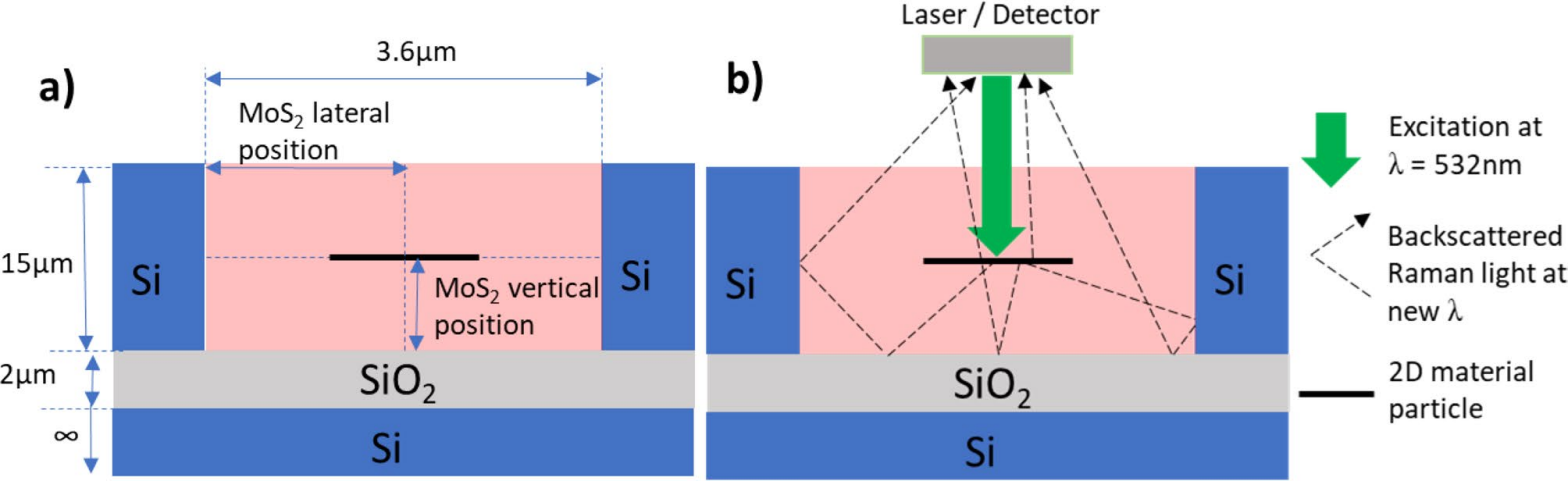
(**a**) Schematic of the microfluidic design showing dimensions. (**b**) Schematic of the experimental setup, showing the incident light and indicative backscattered Raman light pathways.

Let us consider a silicon-on-insulator (SOI) based microfluidic channel (Fig. 1a), with an open top cladding to allow facile in-situ Raman spectroscopy. A silicon substrate (thickness > 1 mm) supports first a buffer layer of silicon dioxide. On this buffer layer is a further layer of silicon. The microfluidic channels are etched into the top silicon layer and terminate at the silicon dioxide boundary. This platform was chosen due to the ease, accuracy, and repeatability of manufacture from a materials standpoint, and the facile manipulation and monitoring of contained liquids owing to the open top cladding. Such channels have previously been demonstrated to enhance the Raman signal intensity^21,26^. In our previous work, similar cavities were designed and used to monitor the position of graphene oxide particles in one dimension^21^. Throughout this work, we will consider the host fluid for the dispersion of particles within the microfluidic channel to be a nematic liquid crystal (specifically, liquid crystal E7). The nematic liquid crystal (LC) host is a birefringent material. However, an advantage of the microfluidic infiltration into SOI cavities is the spontaneously induced planar alignment of the LC through interaction with the surfaces of the Si walls^27^. The LC will therefore have a director which is either parallel or perpendicular to the walls of the channel such that only either the extraordinary or ordinary refractive index is required, rather than some intermediate value. Microfluidic designs were optimised to significantly enhance the back-scattered Raman signal from incorporated particles of molybdenum disulfide (MoS_2_).

To optimise the microfluidic design for facilitating strong confinement of incident light within the channel and to significantly enhance the back-scattered Raman signal ‘emitted’ (Fig. 1b) after interaction with the individual incorporated particles, one can model the variation in the intensity of the Raman bands of the dispersed particles while varying parameters that can be experimentally controlled. Variable parameters in our considered microfluidic layout include, for example, the microfluidic channel width, *w*, the channel depth, *h*, and the buffer oxide (BOX) layer thickness, *h*_*BOX*_. The backscattered Raman signal intensity is numerically determined, using the scattering matrix method (SMM) for wavelengths corresponding to the Raman active bands of the desired (MoS_2_) analyte. Raman scattering itself is a purely quantum mechanical process. There is a random spatial distribution of the photons involved, with no way to accurately predict the emission at the single photon level. However, the optical behaviour of the overall scattered light can still be modelled using a classical electrodynamics approach assuming a large number of Raman scattered photons are emitted. We use the SMM to numerically determine the far-field intensity of the dipole emission from the MoS_2_ analyte that is back-scattered and thus escapes from the microfluidic channel. The use of the SMM allows the simulation of the near- and far-field light distributions for structures with geometries that can be split into clearly-defined layers that are uniform in a minimum of one direction^23–25,28^. The fundamental element of this method is the decomposition of the electric and magnetic fields of light into separate Fourier series in each layer, and subsequently connecting the Fourier components of all adjacent layers in compliance with the boundary conditions of Maxwell’s equations, to give an overall picture of the light propagation within the structure. Analyte particles are modelled as a system of chaotically-oriented oscillating (i.e. the emission direction is randomised) electrical dipoles^20^ within the microfluidic channel, with the specified dipole emission defining the Raman photon emission direction and wavelength. The local components of the electromagnetic field were found, forming material matrices in each layer. By applying an iterative procedure, the total scattering matrix for the whole structure was calculated^29^. The back-scattered Raman intensity was calculated from the components of the scattering matrix. To reach convergence, 801 Fourier harmonics were used. This value was determined by simulating a simplified channel with increasing numbers of Fourier harmonics used, until no change in the result was observed when further increasing the number of harmonics. All numerical analyses were made assuming normal angles of Raman laser incidence and signal collection. The electric field vector of the incident light is oriented parallel to the channel walls (i.e. in the invariant z direction).

The spot size of the Raman laser was effectively considered as equal to the microfluidic cavity width. As the microfluidic channel width was changed in the simulations, the spot size was also considered to change. To account for this, all Raman intensities were normalised by accounting for the incident field strength in the channel. The incident and Raman-scattered wavelengths of the light are considered to be significantly greater than the size of the analyte particles, such that the particle can be considered as a point dipole-an emitter of only Stokes or anti-Stokes photons within the system- and hence the refractive index of the analyte has no effect on the propagation of backscattered light in the cavity. For Fabry-Pérot effects in a layer to be observed experimentally, its thickness should be less than the coherence length of the Raman scattered light^20^. However, this condition is not fulfilled for the silicon substrate layer, hence it is modelled as a semi-infinite material by removing all Fabry-Pérot resonances within the substrate layer.

The microfluidic structures used herein were specifically designed in order to enhance the intensity of the Raman signal from a monolayer MoS_2_ particle positioned at the centre of the channel, for an excitation wavelength of 532 nm. The two characteristic Raman vibrational bands of MoS_2_ are observed at wavenumbers of 386 cm^−1^ (E_2g_) and 404 cm^−1^ (A_1g_) respectively in the case of a monolayer^30,31^. For an excitation wavelength of 532 nm, the scattered radiation will therefore have energies corresponding to wavelengths of 543.15 nm and 543.69 nm, after losing energy to the E_2g_ and A_1g_ vibrational modes of MoS_2_ respectively. By considering the geometry required to maximise the back-scattered intensities of these two wavelengths simultaneously, channels were designed to be etched into a top silicon layer 15 µm deep, with channels spanning 3.6 µm across, and the buffer SiO_2_ layer having a thickness of 2 µm (Fig. 1a).

Using the scattering matrix method again, let us now consider the change in intensity of the measurable Raman signal as a function of the particle's position within the microfluidic channel. Let us now consider a two-dimensional particle (flake) of MoS_2_, parallel to the bottom of the microfluidic channel, with a width of 1 µm. Symmetry allows the representation of the particle to be reduced to a single dipole at the centre. The effect of the MoS_2_ particle’s position on the Raman spectrum signal intensity was modelled by varying the spatial coordinates of the oscillating dipole within the microfluidic channel both laterally (*x*) and vertically (*y*) over a fine grid of positions. As the flake is considered to be 1 µm wide, its centre must be at least 0.5 µm from the wall, giving the *x* position limits. As the flake is considered to essentially be an infinitely thin monolayer, the *y* position limits are the top and bottom of the channel respectively.

**Figure 2:**
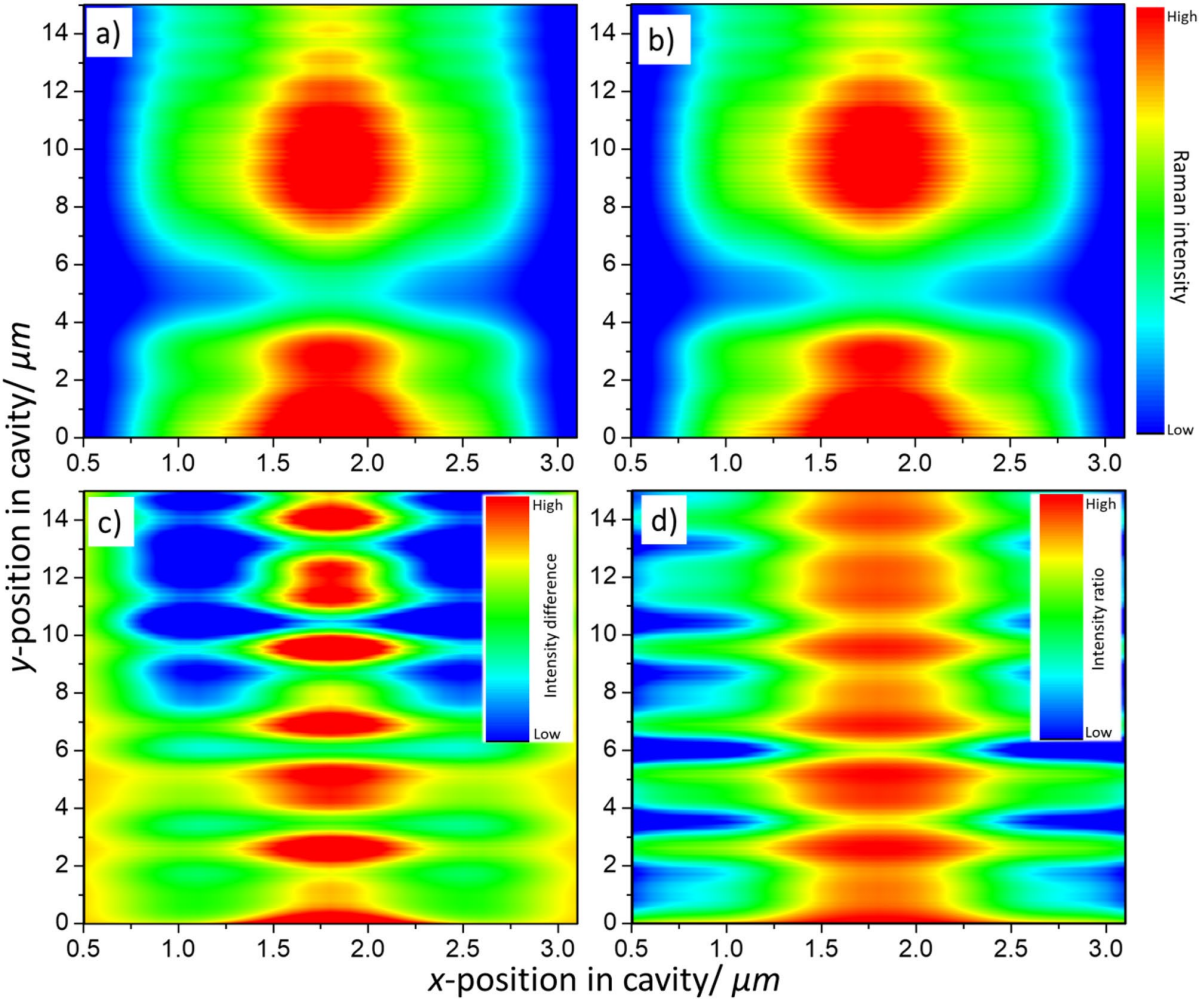
(**a**,**b**) The numerically determined back-scattered intensity for the Raman bands of monolayer MoS_2_ under excitation by a 532 nm laser, with emission corresponding to wavelengths of: (**a**) 543.15 nm for the E_2g_ band and (**b**) 543.69 nm for the A_1g_ band respectively. By comparing experimental spectra to: (**c**) the absolute difference (${I}_{E_{2g}}-{I}_{A_{1g}}$) between the intensities for the two Raman bands of interest, and (**d**) the ratio (${I}_{E_{2g}}/{I}_{A_{1g}}$) between the intensities of the bands resulting from the simulations, one can accurately determine the nanoparticle position.

Very little difference in the Raman intensity is observable at first glance, due to the very small difference in wavelength (${\lambda }_{A_{1g}}-{\lambda }_{E_{2g}}$ = 0.54 nm*,* less than 0.1% of the wavelengths) between the Raman-scattered photons resulting from the interaction of the laser light with each of the two Raman vibrational modes of monolayer MoS_2_ respectively (Fig. 2a,b). However, one can then analyse the absolute difference in the predicted intensities (${I}_{E_{2g}}-{I}_{A_{1g}}$), alongside the ratio (${I}_{E_{2g}}/{I}_{A_{1g}}$) between them, at which point the inter-band variance is more clearly observable (Fig. 2c,d). In our calculations, it is observed that each position of the particle has a unique combination of the ratio and difference of the identities, with the exception of symmetrically equivalent positions. A similar analysis is presented in the supplementary information for graphene oxide (Figures S1, S2).

As test samples, liquid crystalline nanocomposite materials consisting of MoS_2_ flakes homogeneously dispersed in a nematic liquid crystal host (specifically, liquid crystal E7) were synthesised via the following procedure. MoS_2_ was exfoliated from the bulk solid by use of a liquid-phase method, wherein ultrasonication of bulk MoS_2_ particles dispersed in a suitably chosen solvent (chloroform in this case) induces the cleavage of the interlayer van der Waals bonds, with significantly less disruption and damage of the intralayer bonding, in order to give a high aspect ratio of the new, smaller particles formed. While this method produces a solution containing a range of particle shapes and sizes, the vast majority are typically observed to be high aspect ratio platelet shapes with variable numbers of layers. Resulting dispersions of few-layer MoS_2_ were then centrifuged and only an aliquot from the top of the centrifuge tube was used in order to exclude any heavier residual bulk material, or otherwise large MoS_2_ particles. The centrifuged aliquot was then mixed with the commercial nematic liquid crystal formulation (E7) and were subsequently ultrasonicated to ensure homogeneous dispersion of MoS_2_ within the liquid. The organic solvent was then selectively removed from the mixture using a Schlenk vacuum line, due to its lower boiling point than the nematic liquid crystal. This left dispersed, platelet-type MoS_2_ particles suspended in the liquid crystal host. A further ultrasonication was undertaken to ensure the homogeneity of the dispersion. The resultant particle dispersion was then integrated into the designed microfluidic structures, using an infiltration needle to deposit the liquid crystal into an infiltration reservoir and then utilising capillary flow to disperse the fluid into the smaller microfluidic channels. Samples with graphene oxide were produced by a similar method, as described in the supplementary information.

**Figure 3:**
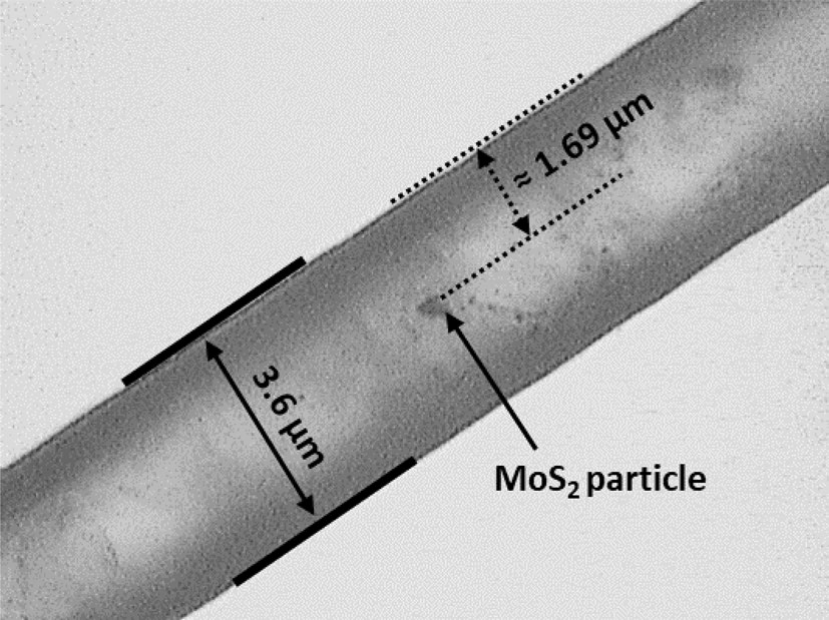
Optical microscopy image of the microfluidic channel under consideration, with a single MoS_2_ particle approximately 1.69 µm (or 1.81 µm) across the 3.6 µm channel.

The experimental Raman spectrum was measured for a particle at an unknown position (Fig. 3) within the microfluidic channel. The peak intensities for the E_2g_ and A_1g_ Raman bands were extracted. The extracted intensities are then normalised against the spectrum for a comparable particle (i.e. a particle with the same number of layers (monolayer)), with the spectrum recorded in an environment where there is negligible influence from the microfluidic channel geometry upon the signal intensity (such as on a bare substrate). Normalisation is necessary here to account for the fact that the intensities of the E_2g_ and A_1g_ Raman bands of MoS_2_ are not expected to be identical—a fact that the scattering matrix method used doesn’t consider—due to the different quantum efficiencies of the underlying interactions between the incident light and the vibrational bands. A further normalisation against the signal from the same microfluidic channel, but without the particle, should also be undertaken to remove any effects not due to the analyte particles—that is, to enable the subtraction of the liquid crystal host spectrum from the MoS_2_ analyte spectrum.

**Figure 4:**
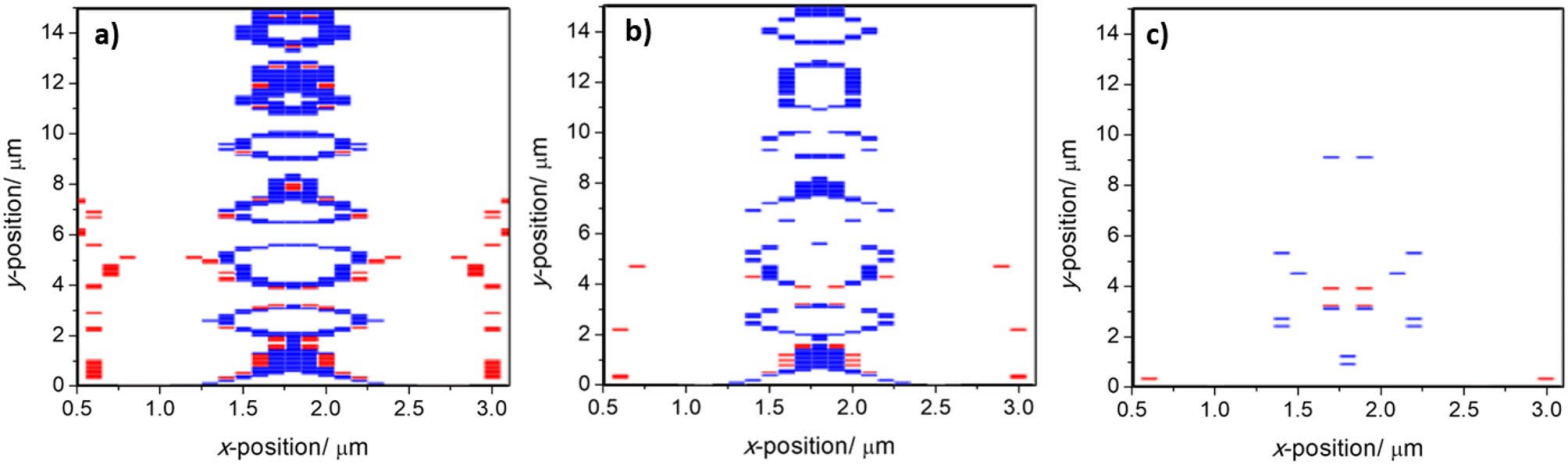
The determination of particle positions by combining scattering matrix method predictions and experimental data for the differences (${I}_{E_{2g}}-{I}_{A_{1g}}$) (red) and ratios (${I}_{E_{2g}}/{I}_{A_{1g}}$) (blue) of the two Raman vibrational bands of MoS_2_ given (**a**) experimental uncertainty of ± 10%, (**b**) ± 5%, and (**c**) ± 2%. The differences (red) have been slightly offset so that the overlap between the two sets of possible positions can be observed.

From the normalised experimental spectrum, the ratio of the peak intensities (${I}_{E_{2g}}/{I}_{A_{1g}}$) is then calculated. The absolute difference between the intensities is also calculated (${I}_{E_{2g}}-{I}_{A_{1g}}$). From the Raman spectrum of the particle in Fig. 3, we obtain ${I}_{E_{2g}}-{I}_{A_{1g}}$ = 0.054 ± 0.002 and ${I}_{E_{2g}}/{I}_{A_{1g}}$ = 1.030 ± 0.002. The values obtained are then compared to those predicted from the numerical SMM calculations (Fig. 4). When doing so we should also account for experimental uncertainty in the Raman spectra. If we consider a rapid scan of the Raman spectrum, with high uncertainty, it is necessary to consider a broad range of values to either side of the exact peak difference or ratio to establish the possible positions of the particle (Fig. 4a), making it hard to identify the precise position as multiple possibilities can be present. By reducing the experimental uncertainty, a corresponding decrease in the number of possibilities is observed (Fig. 4b). It should be noted that, even with low uncertainty in the experimental data, it is likely that the experimental values obtained will lead to the identification of multiple possible positions using either the intensity ratio or intensity difference alone. However, by comparing the possibilities for the position found separately using the peak difference and peak ratios, given sufficient experimental accuracy and precision, a single point of agreement can be found between the possible positions for the particle as determined from the ratio and absolute difference of the peak intensities. This corresponds to the particle's precise position within the channel (Fig. 4b). For the particle in Fig. 3, we obtain an *x* position of either 1.710 or 1.890 µm which compares well with the estimate of 1.69 µm obtained from the optical microscopy image. We also obtain a *y* position of 3.194 µm which cannot be extracted from the optical image. Taking a series of spectra at fixed intervals would then allow the particle movement (or otherwise) to be accurately monitored. Predictions and comparison to optical microscopy for further MoS_2_ particles are shown in the supplementary information (Figures S3). The same process is also followed to determine positions for graphene oxide particles (Figures S4). The results of all position determinations are summarised in Table 1.

**Table 1:** Summary of the particle positions as determined by the method described in this work, with a comparison to the value determined by optical microscopy where possible.

2D Material	Shown in figure	Lateral position				Vertical position
		Our method		Optical microscopy	% Difference	Our method
MoS_2_	3 & 4	1.71	1.89	1.69	1.2	3.194
	S3a & S3d	1.4	2.2	2.11	4.1	13.05
	S3b & S3e	1.39	2.21	1.31	5.8	13.76
	S3c & S3f	1.31	2.29	2.22	3.1	10.9
	S3g & S3j	1.29	2.31	1.38	7.0	8.52
	S3h & S3k	1.19	2.41	2.44	1.2	10.5
	S3i & S3l	1.1	2.5	1.22	10.9	14.82
	S3m & S3n	1.2	2.4	1.3	8.3	13.1
Graphene oxide	S4a–b	1.59	2.01	1.54	3.1	10.45
	S4c–d	0.8	2.8	0.85	6.3	8.78
	S4e–f	1.003	2.597	2.63	1.3	3.51
	S4g–h	0.69	2.91	0.69	0.0	10.5
	S4i–j	1.2	2.4	2.44	1.7	10.45
	S4k–l	0.7	2.9	–	–	3.41

As can be seen, the predictions made using the method described herein show good agreement with direct measurements of the lateral position from optical microscopy. In no case did the difference exceed 11%. It should also be pointed out that there are significant challenges presented by using optical microscopy for measurements. Firstly, the liquid crystal can distort the image, giving a false position. Secondly, depending on how deep in the channel the 2D material particle is, it may not be visible at all. In many cases, it is hard to determine where the edges of the particle are, making it hard to identify the centre of the particle to measure the distance. Overall, these challenges make our method for determining the position superior.

The cavities used were symmetric about *x* = 1.8 µm, giving a Raman signal intensity that also varies symmetrically as a function of position about the same axis. Hence, in this microfluidic geometry, at least two possible positions are determined from any given Raman spectrum, with no way to distinguish between them. The obvious way to solve this in order to give a unique solution is to break the symmetry of the cavity. This could be achieved, for example, by changing the material of one of the cavity’s walls. However, a more practical experimental solution would be to simply realign the Raman laser such that the cavity is asymmetrically illuminated. It is notable also that the procedure described for determining the position in two dimensions simultaneously here is considerably easier to apply than that for a single tracking dimension described in our previous work^21^, where the normalization process required was more challenging. There are two possible factors that limit the accuracy of determining the particle’s position. Firstly, the resolution of the numerically determined data set—there is a minimum uncertainty equivalent to the step between data points calculated by the SMM. Secondly, the accuracy and precision of the obtained experimental spectra. To obtain the position accurately, it is desirable to minimise the signal/noise ratio in the spectrum. There are numerous strategies to achieve this. For instance, longer spectral acquisition times can be used (although this would be at the cost of temporal resolution if tracking a particle). Further optimisation of the signal enhancement due to the cavities would also improve the signal/noise ratio. The key disadvantage of this method is that it is necessary for the particle that is tracked to have two distinguishable Raman peaks. This means that it is not applicable, for example, to hexagonal boron nitride. However, the vast majority of materials fulfil this criterium. One distinct advantage of this methodology is that an individual Raman spectrum can be gathered rapidly; the measurements are only required at a small number of wavelengths (i.e. those corresponding to the Raman-scattered photons of the analyte particle). Therefore it is unnecessary spend time measuring at other wavelengths. In essence, the mechanical properties of the Raman spectrometer itself become the limit the repetition rate of scanning and hence also of the temporal resolution of the positional determination.

## Supplementary information

Supplementary file1

## References

[1] La Spada L, Vegni L (2018). Electromagnetic nanoparticles for sensing and medical diagnostic applications. Materials (Basel).

[2] Patra JK (2018). Nano based drug delivery systems: Recent developments and future prospects. J. Nanobiotechnol..

[3] Galvão C (2018). Antimicrobial coatings from hybrid nanoparticles of biocompatible and antimicrobial polymers. Int. J. Mol. Sci..

[4] Dimov D (2018). Ultrahigh performance nanoengineered graphene-concrete composites for multifunctional applications. Adv. Funct. Mater..

[5] Retterer ST, Melechko A, Hensley DK, Simpson ML, Doktycz MJ (2008). Positional control of catalyst nanoparticles for the synthesis of high density carbon nanofiber arrays. Carbon N. Y..

[6] Jose R, Skačej G, Sastry VSS, Žumer S (2014). Colloidal nanoparticles trapped by liquid-crystal defect lines: A lattice Monte Carlo simulation. Phys. Rev. E.

[7] Ko SH (2007). Direct nanoimprinting of metal nanoparticles for nanoscale electronics fabrication. Nano Lett..

[8] Massé P (2013). Synthesis of size-monodisperse spherical Ag@SiO 2 nanoparticles and 3-D assembly assisted by microfluidics. Langmuir.

[9] Yamamoto H, Ohnuma A, Ohtani B, Kozawa T (2014). Position control of metal nanoparticles by self-assembly. J. Photopolym. Sci. Technol..

[10] Mezzenga R, Ruokolainen J (2009). Nanoparticles in the right place. Nat. Mater..

[11] Jones RR, Hooper DC, Zhang L, Wolverson D, Valev VK (2019). Raman techniques: Fundamentals and frontiers. Nanoscale Res. Lett..

[12] Zhang X, Tan Q-H, Wu J-B, Shi W, Tan P-H (2016). Review on the Raman spectroscopy of different types of layered materials. Nanoscale.

[13] Dies H, Raveendran J, Escobedo C, Docoslis A (2018). Rapid identification and quantification of illicit drugs on nanodendritic surface-enhanced Raman scattering substrates. Sensors Actuators B Chem..

[14] Pilot (2019). A review on surface-enhanced Raman scattering. Biosensors.

[15] Walter A, März A, Schumacher W, Rösch P, Popp J (2011). Towards a fast, high specific and reliable discrimination of bacteria on strain level by means of SERS in a microfluidic device. Lab Chip.

[16] Ackermann KR, Henkel T, Popp J (2007). Quantitative online detection of low-concentrated drugs via a SERS microfluidic system. ChemPhysChem.

[17] Whitesides GM (2006). The origins and the future of microfluidics. Nature.

[18] Kant K, Abalde-Cela S (2018). Surface-enhanced raman scattering spectroscopy and microfluidics: Towards ultrasensitive label-free sensing. Biosensors.

[19] Faneca J, Perova TS, Tolmachev V, Baldycheva A (2018). One-dimensional multi-channel photonic crystal resonators based on silicon-on-insulator with high quality factor. Front. Phys..

[20] Dyakov SA (2011). Optical properties of grooved silicon microstructures: Theory and experiment. J. Exp. Theor. Phys..

[21] Hogan BT (2017). Dynamic in-situ sensing of fluid-dispersed 2D materials integrated on microfluidic Si chip. Sci. Rep..

[22] Tolmachev VA, Baldycheva AV, Berwick K, Perova TS (2012). Influence of fluctuations of the geometrical parameters on the photonic band gaps in one-dimensional photonic crystals. Prog. Electromagn. Res..

[23] Tikhodeev SG, Yablonskii AL, Muljarov EA, Gippius NA, Ishihara T (2002). Quasiguided modes and optical properties of photonic crystal slabs. Phys. Rev. B.

[24] Dyakov SA (2013). Influence of the buffer layer properties on the intensity of Raman scattering of graphene. J. Raman Spectrosc..

[25] Dyakov SA (2012). Surface states in the optical spectra of two-dimensional photonic crystals with various surface terminations. Phys. Rev. B.

[26] Leonardis FD, Passaro VMN (2007). Modelling of Raman amplification in silicon-on-insulator optical microcavities. New J. Phys..

[27] Sengupta A, Herminghaus S, Bahr C (2014). Liquid crystal microfluidics: Surface, elastic and viscous interactions at microscales. Liq. Cryst. Rev..

[28] Christ A, Tikhodeev SG, Gippius NA, Kuhl J, Giessen H (2003). Waveguide-plasmon polaritons: Strong coupling of photonic and electronic resonances in a metallic photonic crystal slab. Phys. Rev. Lett..

[29] Ko DYK, Inkson JC (1988). Matrix method for tunneling in heterostructures: Resonant tunneling in multilayer systems. Phys. Rev. B.

[30] Ramakrishna Matte HSS (2010). MoS2 and WS2 analogues of graphene. Angew. Chemie Int. Ed..

[31] Liang L, Meunier V (2014). First-principles Raman spectra of MoS2, WS2 and their heterostructures. Nanoscale.

