# Supplementary material for: Spatial tracking of individual fluid dispersed particles via Raman spectroscopy
Source: Sci Rep. 2020 Sep 1;10:14350. doi: 10.1038/s41598-020-71253-x (PMC7463031; doi:10.1038/s41598-020-71253-x)
Supplement: Supplementary file 1 — Supplementary file1 [file 41598_2020_71253_MOESM1_ESM.docx]

SUPPLEMENTARY INFORMATION for “Spatial tracking of individual fluid dispersed particles *via* Raman spectroscopy”

Benjamin Thomas Hogan ^1,2,3^*, Jennifer O’Dowd ^1,4^, Joaquin Faneca Ruedas ^1,2^, Alexander Baranov ^5^ and Anna Baldycheva ^1,5^.

^1^ Department of Engineering, University of Exeter, Exeter, United Kingdom

^2^ EPSRC Centre for Doctoral Training in Metamaterials, University of Exeter, Exeter, United Kingdom

^3^ Department of Information Technology and Electrical Engineering, University of Oulu, Finland

^4^ Department of Engineering, University of Cambridge, Cambridge, United Kingdom

^5^ ITMO University, Saint Petersburg 197101, Russia

***** Correspondence: [bh341@exeter.ac.uk](mailto:bh341@exeter.ac.uk)

For simplicity, we use the same chip structure as used for measurements of MoS_2_. The structure is shown schematically in Figure S1. While this structure is not optimised for detecting graphene oxide (GO), the signal is still sufficient.


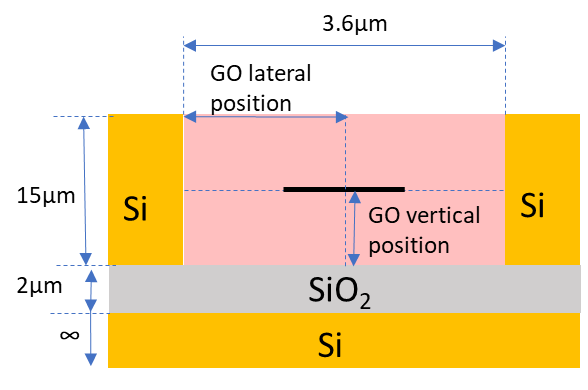


**Figure S1:** Schematic of the microfluidic cavity design showing dimensions for graphene oxide simulations and experiments.

We use the scattering matrix as before to generate the predicted position dependence of the Raman spectrum of GO within the cavity (Figure S2). We make the same assumptions as for MoS_2_. However, in this case the Raman bands are expected at 574.15 nm and 579.14 nm for the D and G bands respectively (corresponding to Raman shifts of 1345 cm^-1^ and 1600 cm^-1^ respectively) ^1^.

**Figure S2**: **a-b)** The numerically determined back-scattered intensity for the Raman bands of graphene oxide under excitation by a 532 nm laser, with emission corresponding to wavelengths of: **a)** 579.14 nm (G band) **b)** 574.15 nm (D band) respectively. By comparing experimental spectra to: **c) t**he absolute difference ($I_{G}-I_{D}$) between the intensities for the two Raman bands of interest, and **d)** the ratio ($I_{G}/I_{D}$) between the intensities of the bands resulting from the simulations, one can accurately determine the nanoparticle position.


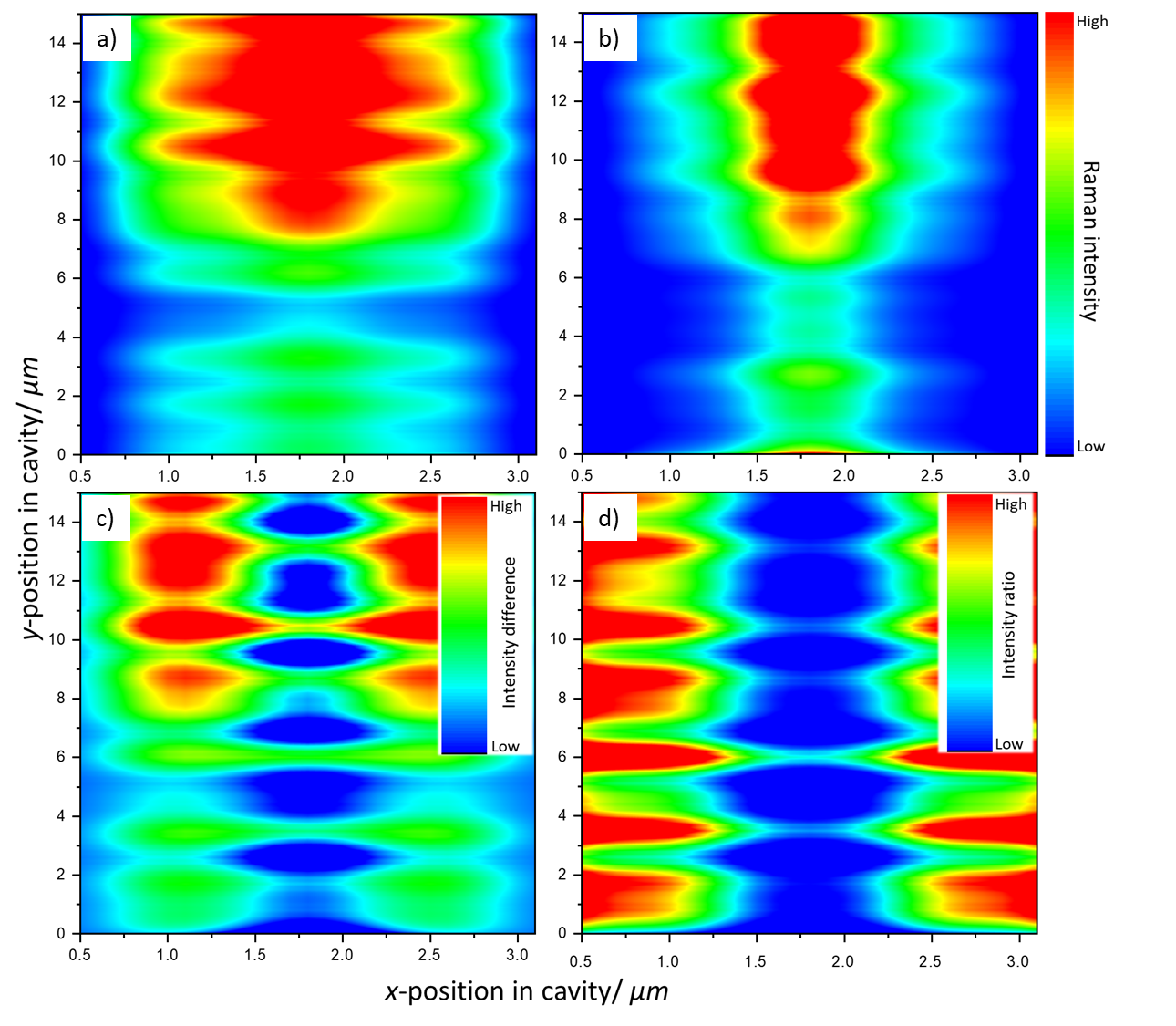


Additional MoS_2_ particles were tracked by the same method as described for the MoS_2 ­_particle in figures 3 and 4 in the main text. The results are shown in Figure S3, and summarised in Table 1. Estimations of positions *via* optical microscopy can present significant challenges. Note how in many of the optical microscopy images, the edges of the channel can be poorly defined (e.g. Fig. S3b,c,g,h,I,m). Thus it is hard to determine a lateral displacement accurately. In some cases (Fig. S3c) it can be hard to identify a single particle of interest. In further cases, there is little contrast between the particle and the background (e.g. Fig. S3a,g,m), such that identifying the edges and centre of the particle is challenging. However, all particles can be successfully tracked from their Raman spectra, and the results match well with estimates made from the optical images.


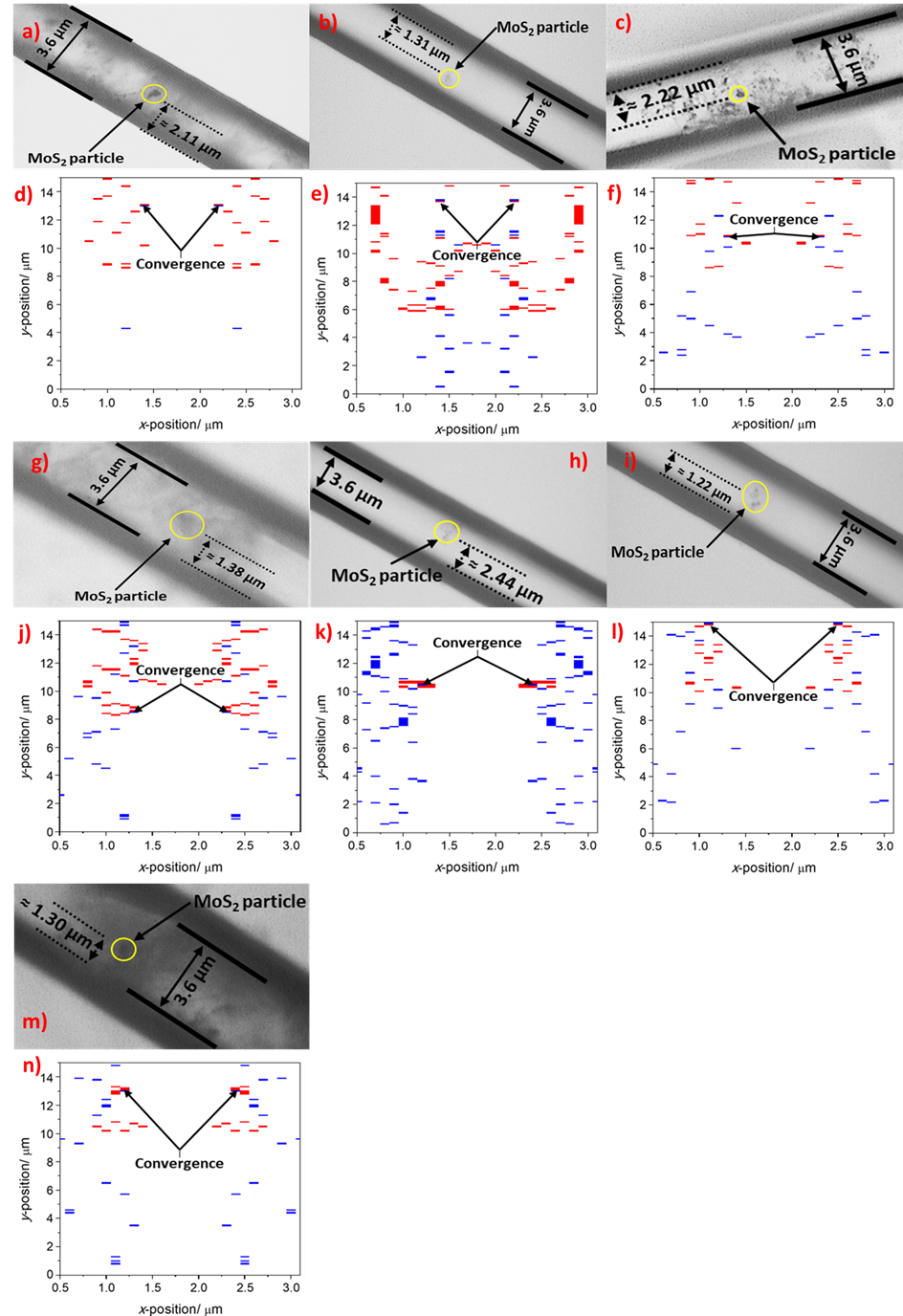


**Figure S3**: Optical microscopy images of different MoS_2_ particles and their corresponding positions in two dimensions as determined from Raman spectrum intensities. Positions are determined from the Raman spectra using the intensity differences (red) and ratios (blue). The convergence points of the separate predictions made using the differences and ratios are marked by the black arrows. Optical microscopy images show an estimated lateral displacement. The particle shown in **a)** is tracked in **d)**. The particle shown in **b)** is tracked in **e)**. The particle shown in **c)** is tracked in **f)**. The particle shown in **g)** is tracked in **j)**. The particle shown in **h)** is tracked in **k)**. The particle shown in **i)** is tracked in **l)**. The particle shown in **m)** is tracked in **n)**. The precise tracking result values are given in Table 1.

For GO experiments, liquid crystalline nanocomposite materials consisting of GO flakes homogeneously dispersed in liquid crystal E7 were synthesised via the following procedure. GO was exfoliated from the bulk solid by use of a liquid-phase method, wherein ultrasonication of bulk GO particles dispersed in a suitably chosen solvent (deionised water in this case) induces the cleavage of the interlayer van der Waals bonds. Resulting dispersions of few-layer GO were then centrifuged and only an aliquot from the top of the centrifuge tube was used in order to exclude any heavier residual bulk material, or otherwise large GO particles. The centrifuged aliquot was then dried to remove the water, and redispersed in chloroform. This redispersion was then mixed with the liquid crystal, and subsequently ultrasonicated to ensure homogeneous dispersion of GO within the liquid crystal. The chloroform was then selectively removed from the mixture using a Schlenk vacuum line, due to its lower boiling point than the nematic liquid crystal. This left dispersed, platelet-type GO particles suspended in the liquid crystal host. A further ultrasonication was undertaken to ensure the homogeneity of the dispersion. The resultant particle dispersion was then integrated into the designed microfluidic structures, using an infiltration needle to deposit the liquid crystal into an infiltration reservoir and then utilising capillary flow to disperse the fluid into the smaller microfluidic channels.

**Figure S4**: Optical microscopy images of different GO particles and their corresponding positions in two dimensions as determined from Raman spectrum intensities. Positions are determined from the Raman spectra using the intensity differences (red) and ratios (blue). The convergence points of the separate predictions made using the differences and ratios are marked by the black arrows. Optical microscopy images show an estimated lateral displacement. The particle shown in **a)** is tracked in **b)**. The particle shown in **c)** is tracked in **d)**. The particle shown in **e)** is tracked in **f)**. The particle shown in **g)** is tracked in **h)**. The particle shown in **i)** is tracked in **j)**. The particle shown in **k)** is tracked in **l)**. The precise tracking result values are given in Table 1.


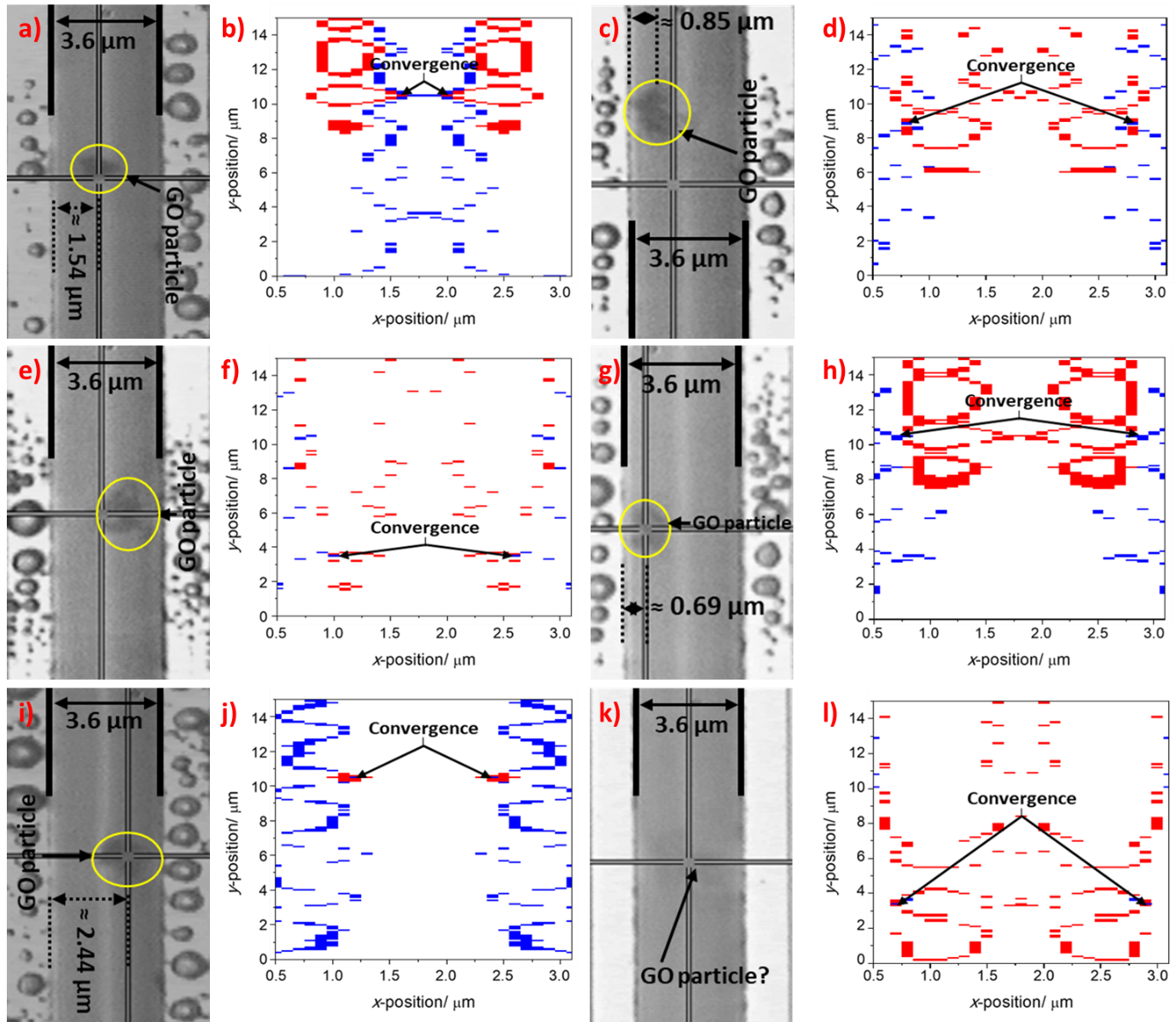


GO particles were tracked by the same method as described for MoS_2_, and the results are presented in Figure S4 and summarised in Table 1. Of particular note is the particle shown in Fig. S4k and tracked in Fig. S4l. This particle is not visible using optical microscopy but can clearly be tracked using its Raman spectrum.

**Supplementary Reference:**

1. Hogan, B. T. *et al.* Dynamic in-situ sensing of fluid-dispersed 2D materials integrated on microfluidic Si chip. *Sci. Rep.* **7**, 42120 (2017).
